# Spinal movement disorders in NMOSD, MOGAD, and idiopathic transverse myelitis: a prospective observational study

**DOI:** 10.1007/s00415-024-12527-6

**Published:** 2024-07-08

**Authors:** Hesham Abboud, Rongyi Sun, Nikhil Modak, Mohamed Elkasaby, Alexander Wang, Michael Levy

**Affiliations:** 1 Multiple Sclerosis and Neuroimmunology Program, University Hospitals Cleveland Medical Center, Bolwell, 5th Floor, 11100 Euclid Avenue, Cleveland, OH 44106 USA; 2 Parkinson’s and Movement Disorders Center, University Hospitals Cleveland Medical Center, Cleveland, OH USA; 3 Case Western Reserve University School of Medicine, Cleveland, OH USA; 4 Division of Neuroimmunology and Neuroinfectious Diseases, Massachusetts General Hospital, Boston, MA USA

**Keywords:** NMOSD, MOGAD, Myelitis, Movement disorders, Tonic spasms, Dystonia, Spinal

## Abstract

**Background:**

Retrospective studies suggest that spinal movement disorders, especially tonic spasms, are prevalent in NMOSD. However, there have been no prospective studies evaluating spinal movement disorders in NMOSD, MOGAD, and idiopathic transverse myelitis (ITM).

**Methods:**

Patients referred to a tertiary neuroimmunology clinic for spinal cord demyelination (excluding MS) were evaluated. All patients answered a movement disorders survey and underwent a movement disorder-focused exam. Movement disorders were compared among patients with NMOSD with and without AQP4-IgG, MOGAD, and ITM. Patients with and without involuntary movements were also compared to identify predictors of spinal movement disorders.

**Results:**

Sixty-three patients were evaluated from 2017 to 2021 (71% females, median age 49 years, range 18–72 years, median disease duration 12 months, range 1–408). Of the total, 49% had ITM, 21% had NMOSD without AQP4-IgG, 19% had NMOSD with AQP4-IgG, and 11% had MOGAD. Movement disorders were present in 73% of the total patients and were most frequent in NMOSD with AQP4-IgG (92%) and least frequent in MOGAD (57%). The most frequent spinal movement disorders were tonic spasms (57%), focal dystonia (25%), spinal tremor (16%), spontaneous clonus (9.5%), secondary restless limb syndrome (9.5%), and spinal myoclonus (8%). Multivariate analysis showed that longitudinally extensive myelitis and AQP4-IgG are independent risk factors for the development of spinal movement disorders, while MOG-IgG and African American race were associated with a lower risk of developing these movement disorders.

**Conclusions:**

Spinal movement disorders are highly prevalent in non-MS demyelinating disorders of the spinal cord. Prevalence rates exceed those reported in MS and retrospective NMOSD studies.

**Supplementary Information:**

The online version contains supplementary material available at 10.1007/s00415-024-12527-6.

## Introduction

Movement disorders secondary to spinal cord demyelination are the most frequent movement disorders in multiple sclerosis (MS) [1]. Retrospective studies and case series suggest that spinal movement disorders, especially tonic spasms, are even more prevalent in neuromyelitis optica spectrum disorder (NMOSD) [2–5]. This is likely related to the more extensive inflammation and involvement of the motor pathways in NMOSD patients compared to MS patients [6]. We previously conducted a retrospective study of spinal movement disorders in NMOSD patients [7] and a prospective study of all movement disorders in an MS-predominant cohort [1]. However, there have been no prospective studies evaluating spinal movement disorders in NMOSD. In addition, spinal movement disorders have not been evaluated in patients with myelin oligodendrocyte glycoprotein antibody disease (MOGAD) and idiopathic transverse myelitis (ITM). Both MOGAD and ITM share similar features with NMOSD that are different from MS, including longitudinally extensive transverse myelitis (LETM, as defined by a spinal lesion extending over three or more vertebral levels), more severe attacks, and a higher frequency of motor and sphincteric involvement [8, 9]. There are also important differences between these three conditions, including the underlying immunopathology, response to treatment, and recurrence rates [10]. Moreover, clinical and radiological differences exist even within the NMOSD category depending on aquaporin-4 antibody (AQP4-IgG) serostatus [11]. In this study, we aimed to prospectively evaluate spinal movement disorders in patients with NMOSD, MOGAD, and ITM focusing on prevalence and phenotypic stratification. A second goal was to evaluate predictors of spinal movement disorders in patients with spinal cord demyelination.

## Methods

We conducted a prospective study at the neuroimmunology clinic of University Hospitals Cleveland Medical Center from 2017 to 2021. Patients were included if they were diagnosed with NMOSD (with or without AQP4-IgG according to the 2015 International consensus criteria) [12], or MOGAD (demyelinating disease with positive MOG antibody by cell-based assay excluding false positive cases), or ITM (according to the 2002 transverse myelitis consortium diagnostic criteria, including relapsing cases + negative AQP4 and MOG antibodies) [13]. Although the study was conducted prior to the publication of 2023 International MOGAD panel diagnostic criteria, the criteria were applied retrospectively to the MOGAD patients and all fulfilled the new criteria [9]. All patients were tested for AQP4 and MOG antibodies by cell-based assay at the Mayo Clinic Laboratory. Patients were evaluated by a neurologist trained in both neuroimmunology and movement disorders. Each patient answered a movement disorder survey and underwent a movement disorder-focused exam conducted by the principal investigator. The movement disorder exam was based on a modification of the Unified Parkinson Disease Rating Scale (UPDRS). The movement disorder survey and exam were integrated into standard clinical visits. As part of an ongoing prospective study of movement disorders in neuroimmunological diseases, anonymized video samples of the different phenomenologies found on exam were recorded. Video samples were then rated for phenomenology by two independent movement disorder specialists blinded to patient history and diagnosis. Patients were excluded if they lacked clinical and/or radiological evidence of current or prior myelitis at the time of study enrollment or if they were deemed to have myelitis secondary to a systemic inflammatory disease (e.g., neurosarcoidosis). All patients underwent spinal cord imaging.

Spinal movement disorders were defined according to previously published criteria (Table 1) [7] and were compared between patients with NMOSD with AQP4-IgG, NMOSD without AQP4-IgG, MOGAD, and ITM. We also compared patients with and without spinal movement disorders to identify predictors of spinal movement disorders in patients with myelitis.

**Table 1:** Proposed classification and definitions of spinal movement disorders

Spinal movement disorder	Definition
**Spinal dystonia**	Sustained muscle contraction of antagonistic muscles resulting in complex abnormal posture (other than simple flexion, extension, or adduction) that can be patterned, twisting, and may be tremulous, secondary to spinal cord pathology. Spinal dystonia is typically diagnosed in absence of concomitant basal ganglia pathology
Paroxysmal focal dystonia	Paroxysmal involuntary sustained muscle contraction of antagonistic muscle groups resulting in abnormal posture (other than simple flexion, extension, or adduction) secondary to spinal cord pathology
Non-paroxysmal (fixed) focal dystonia	Persistent (non-paroxysmal) sustained muscle contraction of antagonistic muscle groups resulting in fixed abnormal posture secondary to spinal cord pathology
Spinal hemi-dystonia	Fixed (or rarely paroxysmal) involuntary sustained muscle contraction of antagonistic muscle groups resulting in abnormal posture of the hemi-body (upper limb, lower limb, trunk) secondary to spinal cord pathology
**Tonic spasms**	Paroxysmal sustained increase in muscle tone involving one set of muscle groups leading to isometric contraction or simple posturing in flexion, extension or adduction secondary to spinal cord pathology
Flexor tonic spasm	Paroxysmal sustained increase in muscle tone resulting in visible tonic posturing of the affected body part (often the whole limb or part of the limb) in flexion secondary to spinal cord pathology
Extensor tonic spasm	Paroxysmal sustained increase in muscle tone resulting in visible tonic posturing of the affected body part (often the whole limb or part of the limb) in extension secondary to spinal cord pathology
Adductor tonic spasm	Paroxysmal sustained increase in muscle tone resulting in visible tonic posturing of the affected body part (often the whole limb or part of the limb) in adduction (or inversion) secondary to spinal cord pathology
Isometric tonic spasm	Paroxysmal sustained increase in muscle tone that can be felt by the patient and palpated by the examiner but does not result in visible change in posture (e.g., abdominal wall muscles) secondary to spinal cord pathology
Complex tonic spasm	A combination of two or more tonic spasms of combined phenomenology affecting two or more adjacent or distant body parts
**Spinal myoclonus**	Sudden, brief (non-sustained) shock-like muscle contraction that can be focal, segmental or propriospinal secondary to spinal cord pathology
Focal/segmental spinal myoclonus	Sudden, brief (non-sustained), shock-like focal muscle contraction of one or more adjacent body parts secondary to spinal cord pathology
Propriospinal myoclonus	Sudden, brief, shock-like, arrhythmic jerks of the trunk, hips, and knees (sparing the head) in flexion or extension secondary to spinal cord pathology
**Spinal movement disorders associated with sensory phenomenon**	Abnormal movement triggered by sensory changes in the affected limb secondary to spinal cord pathology
Secondary restless limb syndrome	Unpleasant or uncomfortable urge to move the limb during periods of inactivity that is transiently relieved by movement, secondary to spinal cord pathology
Painful legs moving toes syndrome	Involuntary repetitive, non-rhythmic movement of the toes in vertical or horizontal planes associated with painful sensation in the legs/feet secondary to spinal cord pathology
Pseudoathetosis	Slow, sinuous, writhing movement of the distal limbs due to deep sensory deprivation impairing joint position sense, often aggravated by eye closure or visual distraction, secondary to spinal cord pathology involving the posterior column
**Spinal tremor**	Postural, kinetic, and/or orthostatic tremor (involuntary, rhythmic, oscillatory movement) due to interruption of the spinocerebellar tracts secondary to spinal cord pathology
Spinal action tremor	Postural and/or kinetic tremor occurring after a spinal cord pathological event involving the spinocerebellar tracts in absence of brainstem, cerebellar, or ganglionic lesions
Spinal orthostatic tremor	Fast (13–18 Hz) tremor of the legs while standing that is relieved by sitting or walking, and is often palpable rather than visible, secondary to spinal cord pathology
**Spontaneous clonus**	Spontaneous (non-induced) involuntary, rhythmic muscle contractions and relaxations associated with spasticity secondary to spinal cord pathology. It commonly involves the ankle or the wrist and appears in certain positions or with certain active movements

This study was approved by the institutional review board of University Hospitals Cleveland Medical Center. A written informed consent was obtained from patients in whom identifiable videos were obtained. Source data can be made available for any qualified investigator.

### Statistical analysis

Descriptive statistics were used to summarize the data (i.e., counts, percentages, means, medians, ranges, and standard deviations). Categorical variables were compared using Pearson *χ*^2^ or Fisher's exact test, and continuous variables were compared using independent *t* test or Welch *t* test. Because of the hypothesis-generating nature of the comparison between disease states, no correction for multiple comparisons was performed. Multivariate logistic regression was conducted to identify independent predictors for development of spinal movement disorders. All tests were two-tailed, and the alpha was set at 0.05. Cohen’s Kappa was calculated for interrater agreement. All data were processed through IBM SPSS Statistics 28.0 for Windows (SPSS Inc., Chicago, IL, USA).

## Results

Eighty patients were evaluated from 2017 to 2021. Seventeen NMOSD/MOGAD patients without spinal cord lesions were excluded. The remaining 63 patients were analyzed (71% females, median age 49 years, range 18–72 years, median disease duration 12 months, range 1–408). Of the total, 31 (49%) had ITM, 13 (21%) had NMOSD without AQP4-IgG, 12 (19%) had NMOSD with AQP4-IgG, and seven (11%) had MOGAD. Table 2 compares clinical and demographic data among the four groups of patients. Spinal MRI showed LETM in 36 (57%) patients. Basal ganglia and cortical lesions were absent except in one patient each. Thirteen patients (21%) had brainstem lesions mostly in the form of extension of high cervical myelitis into the lower medulla oblongata (only three patients with isolated brainstem lesions without high cervical involvement).

**Table 2:** clinical and demographic data of the entire cohort by diagnosis

	All patients (*n* = 63)	NMOSD with AQP4-IgG (*n* = 12, 19.0%)	NMOSD without AQP4-IgG (*n* = 13, 20.6%)	MOGAD (*n* = 7, 11.1%)	ITM (*n* = 31, 49.2%)
Age	48.32 ± 13.8	51.0 ± 18.4	44.6 ± 14.0	48.7 ± 11.3	48.7 ± 12.6
Sex					
Male	18 (28.6)	0 (0)	2 (15.4)	3 (42.9)	13 (41.9)
Female	45 (71.4)	12 (100)	11 (84.6)	4 (57.1)	18 (58.1)
Ethnicity					
White	42 (66.7)	3 (25.0)	9 (69.2)	4 (57.1)	26 (83.9)
African American	19 (30.2)	8 (66.7)	3 (23.1)	3 (42.9)	5 (16.1)
Other	2 (3.2)	1 (8.3)	1 (7.7)	0 (0)	0 (0)
Disease duration (months)	54.7 ± 96.7	78.7 ± 124.9	81.38 ± 108.4	66.9 ± 126.9	31.5 ± 67.6
Follow up duration (months)	13.5 ± 13.8	17.2 ± 14.8	24.15 ± 17.7	14.43 ± 13.0	7.42 ± 8.2
Cervical lesion	45 (71.4)	10 (83.3)	9 (69.2)	5 (71.4)	21 (67.7)
Thoracic lesion	40 (63.5)	9 (75.0)	10 (76.9)	5 (71.4)	16 (53.3)
Conus involvement	4 (6.3)	1 (8.3)	2 (15.4)	0 (0)	1 (3.3)
Basal ganglia lesion	1 (1.5)	1 (8.3)	0 (0)	0 (0)	0 (0)
Brainstem lesion	13 (21)	4 (33)	4 (31)	4 (57)	2 (6)
Cortical lesion	1 (1.5)	0 (0)	0 (0)	1 (14)	0 (0)
Any movement disorder	46 (73.0)	11 (91.7)	10 (76.9)	4 (57.1)	21 (67.7)
Disease/relapse presentation	7 (15.2)	0 (0)	1 (10.0)	2 (50)	5/21 (23.8)
Post-relapse	43 (93.5)	11 (100)	9 (90.0)	2 (50)	21/21 (100)
Total FTS	6 (9.5)	3 (25)	1 (7.6)	0 (0)	2 (6.5)
Total ETS	9 (14.2)	2 (16.7)	2 (15.3)	0 (0)	5 (16.1)
Total ATS	3 (4.7)	1 (8.3)	0 (0.0)	0 (0)	2 (6.5)
Total ITS	28 (44.4)	9 (75.0)	6 (46.2)	0 (0)	13 (41.9)
Total CTS	1 (1.6)	0 (0.0)	1 (7.6)	0 (0)	0 (0.0)
Total tonic spasms	36 (57.1)	10 (83.3)	9 (69.2)	0 (0)	17 (54.8)
Total dystonia	16 (25.4)	3 (25.0)	5 (38.5)	1 (14.3)	7 (22.5)
Total clonus	6 (9.5)	1 (8.3)	0 (0.0)	1 (14.3)	4 (12.9)
Total tremor	10 (15.8)	4 (33.3)	1 (7.6)	2 (28.6)	3 (9.7)
Total RLS	6 (9.5)	0 (0.0)	1 (7.6)	0 (0.0)	4 (12.9)
Total myoclonus	5 (7.9)	2 (16.7)	2 (15.3)	0 (0.0)	1 (3.2)
Spasticity	23 (36.5)	4 (33.3)	6 (46.2)	2 (28.6)	11 (35.5)
Other movement disorders	3 (4.7)	0 (0)	1 (7.6)	0 (0)	2 (6.5)
Painful SMDs	16 (25.3)	7 (58.3)	5 (38.5)	0 (0)	4 (12.9)
LETM	36 (57.1)	10 (83.3)	8 (61.5)	5 (71.4)	13 (41.9)
Anterior cord lesion	21 (33.3)	7 (58.3)	6 (46.2)	3 (42.8)	5 (16.1)
Posterior cord lesion	22 (34.9)	6 (50.0)	6 (46.2)	1 (14.3)	9 (29.0)
Lateral cord lesion	14 (22.2)	6 (50.0)	5 (38.5)	1 (14.3)	2 (6.5)
Central cord lesion	19 (30.2)	6 (50.0)	7 (53.8)	1 (14.3)	5 (16.1)

*FTS* flexor tonic spasms, *ETS* extensor tonic spasms, *ATS* adductor tonic spasms, *ITS* isometric tonic spasms, *CTS* complex tonic spasms, *RLS* restless limb syndrome, *SMDs* spinal movement disorders, *LETM* longitudinally extensive transverse myelitis

### Spinal movement disorders prevalence and subtypes

**Fig. 1:**
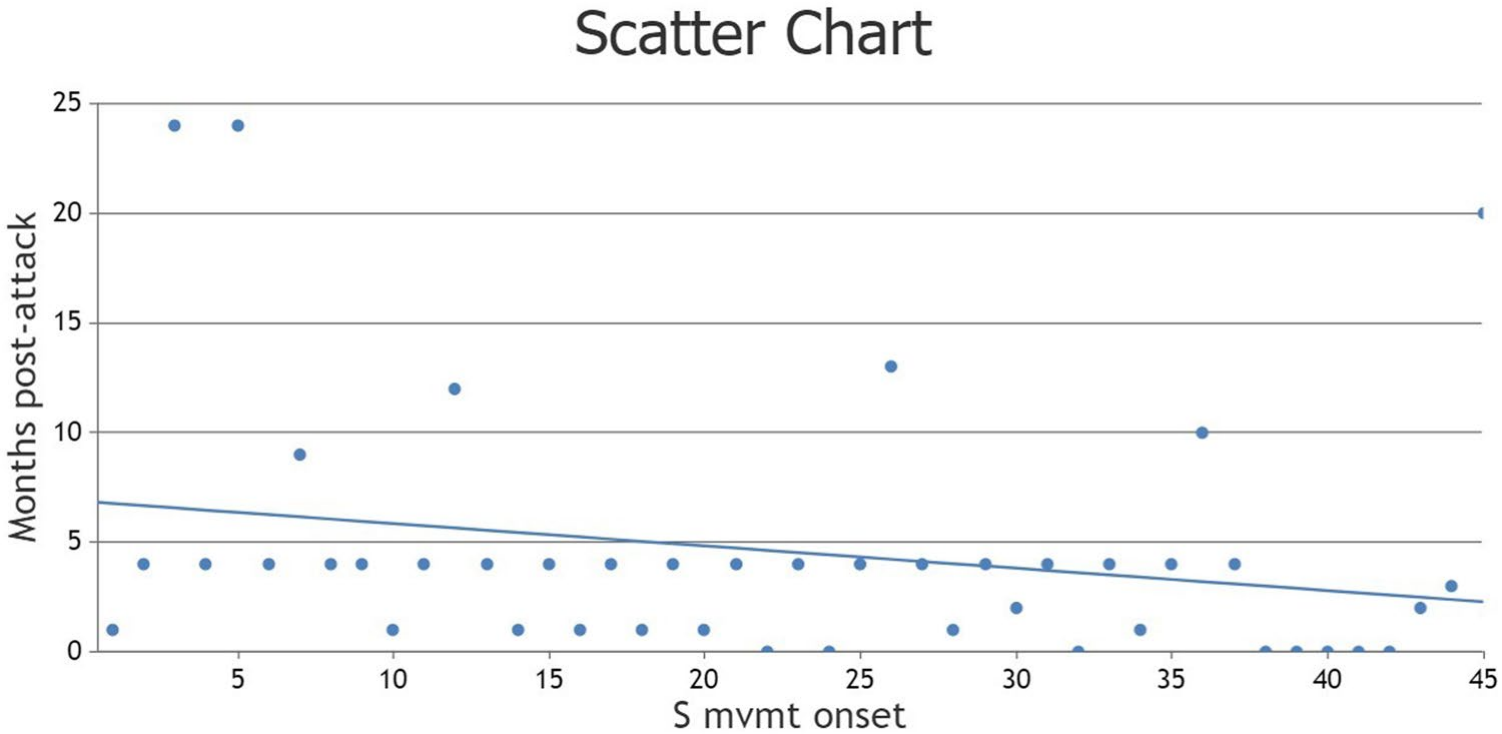
Scatter plot showing the interval (in months) between the index spinal relapse and the onset of spinal movement disorders of the study cohort

Spinal movement disorders were present in 46 (73%) patients and were most frequent in NMOSD with AQP4-IgG (11 patients, 92%), followed by NMOSD without AQP4-IgG (10 patients, 77%), ITM (21 patients, 68%), and least frequent in MOGAD (4 patients, 57%). Spinal movement disorders were the first symptom of the disease heralding the initial relapse in 7 (11%) patients (mostly tonic spasms occurring simultaneously or shortly before the onset of motor weakness). Otherwise, in most cases (93.5%), spinal movement disorders started during recovery from relapse with an average interval of 4.7 months post-relapse (Fig. 1). Some patients who initially presented with tonic spasms in their first relapse later developed other spinal movement disorder phenotypes in the post-relapse phase (hence, the percentages 11% + 93.5% are higher than 100%). Seventeen patients developed new spinal movement disorders during the study period that were not present at the time of initial evaluation concordant with an incidence rate of 0.07 per patient-year. The most frequent spinal movement disorders were tonic spasms (36 patients, 57%), focal dystonia (16, 25%), spinal tremor (10, 16%), spontaneous clonus (6, 9.5%), secondary restless limb syndrome (RLS) (6, 9.5%), and spinal myoclonus (5, 8%). Videos 1–4 show demonstrative examples of spinal movement disorders from this cohort. Tonic spasms were most commonly isometric (77% of all tonic spasms), followed by extensor (25%), flexor (17%), adductor (8%), and complex (3%) spasms. Dystonia was fixed in 52% and paroxysmal in 48% of the total patients with dystonia. Tremor was postural and/or kinetic in nature in all patients mostly involving the hands. Two patients had a resting component in addition to the action component. One of them had Holmes-like tremor involving the upper and lower extremity in the presence of a continuous lesion involving the upper cervical cord and lower brainstem (this patient was previously reported) [14]. Four patients experienced transient postural tremors while on corticosteroids that resolved once steroid therapy was stopped, and were not counted as spinal movement disorders. There were no cases of acquired orthostatic tremor in this cohort. Myoclonus was focal in all patients. There were no cases of propriospinal myoclonus in this cohort. RLS was rare and there were no other cases of spinal movement disorders associated with sensory phenomenon in this cohort, including no cases of pseudoathetosis. The agreement rate of the two blinded video raters on phenomenology was 93% (Cohen’s Kappa = 0.864, standard error = 0.076, CI 0.715–1.0) and disagreement was resolved by consultation with the principal investigator.

The lower extremities were affected in 91% of all patients with spinal movement disorders manifesting all movement phenotypes. Extensor tonic spasms and RLS were exclusively seen in the lower extremities. The upper extremities were involved in 43% of all patients with spinal movement disorders, mainly manifesting as spinal tremor, focal dystonia, and/or flexor/isometric tonic spasms. Axial muscles were involved in 28% of patients mostly manifesting as isometric tonic spasms. One patient had paroxysmal laterocollis.

One or more spinal movement disorders were found on exam during the study period in 31 patients (67%). In the remaining patients, spinal movement disorders were confirmed by history and movement disorder survey. Spinal spasticity was present on exam in 36% of the entire cohort, and some patients with spinal movement disorders, including some with tonic spasms, did not have spasticity on formal exam (indicating that these patients had isolated phasic spasticity only manifesting as spasms).

Apart from fixed dystonia, all other spinal movement disorders were paroxysmal or intermittent (80%). The most common triggers of the paroxysmal involuntary movements included movement/transfers (kinesigenic spinal movement disorders) in 19.5% of patients, rest/night-time (19.5%), cold weather (6.5%), tactile stimulation (4%), and bowel movements (2%). Fifteen percent of patients reported that their spinal movement disorders occurred spontaneously without known triggers.

Only 25% of the reported spinal movement disorders were painful and pain was exclusive to tonic spasms (especially isometric) and paroxysmal dystonia. All other spinal movement disorders were painless. Over half the patients with tonic spasms reported that their spasms were painless.

In addition to pain, spinal movement disorders had several other impacts, including interference with ambulation (15% of patients with movement disorders), negative impact on sleep (4%), impairment of skin hygiene (4%), triggering falls (2%), and negative impact on driving (2%).

Most patients (59%) were treated with baclofen and/or tizanidine for their spinal movement disorders, while botulinum toxin injections were used in 15% and carbamazepine/oxcarbazepine in 11%. Some of these medications were also used to address spasticity. Most patients were on gabapentin for neuropathic pain and to also help with spasms. Nine patients (19.5%) experienced complete resolution of their spinal movement disorders after an average symptomatic duration of 11 months (range 6–24). In most patients, resolution of spinal movement disorders occurred spontaneously over time but one patient each reported complete response to carbamazepine, baclofen, and corticosteroids. Partial improvement was reported by 37% of patients mostly in response to muscle relaxants (baclofen and/or tizanidine) and to a lesser degree in response to carbamazepine, oxcarbazepine, or botulinum toxin injections. Persistent or worsening spinal movement disorders were reported by 43% of all patients with spinal movement disorders during the study period despite various therapeutic trials with different symptomatic medication classes.

### Comparison of spinal movement disorders by diagnosis

Patients with NMOSD with or without AQP4-IgG, compared to all other patients were more likely to have LETM (*P* = 0.041, RR 1.542, 95% CI [1.030–2.308]), anterior spinal lesions (*P* = 0.009, RR 2.473, 95% CI [1.220–5.011]), lateral spinal lesions (*P* = 0.000, RR 5.580, 95% CI [1.744–17.855]), or central spinal lesions (*P* = 0.002, RR 3.297, 95% CI [1.464–7.425]). Unexpectedly, three NMOSD patients had conus involvement as opposed to one ITM patient and none of the MOGAD patients.

**Fig. 2:**
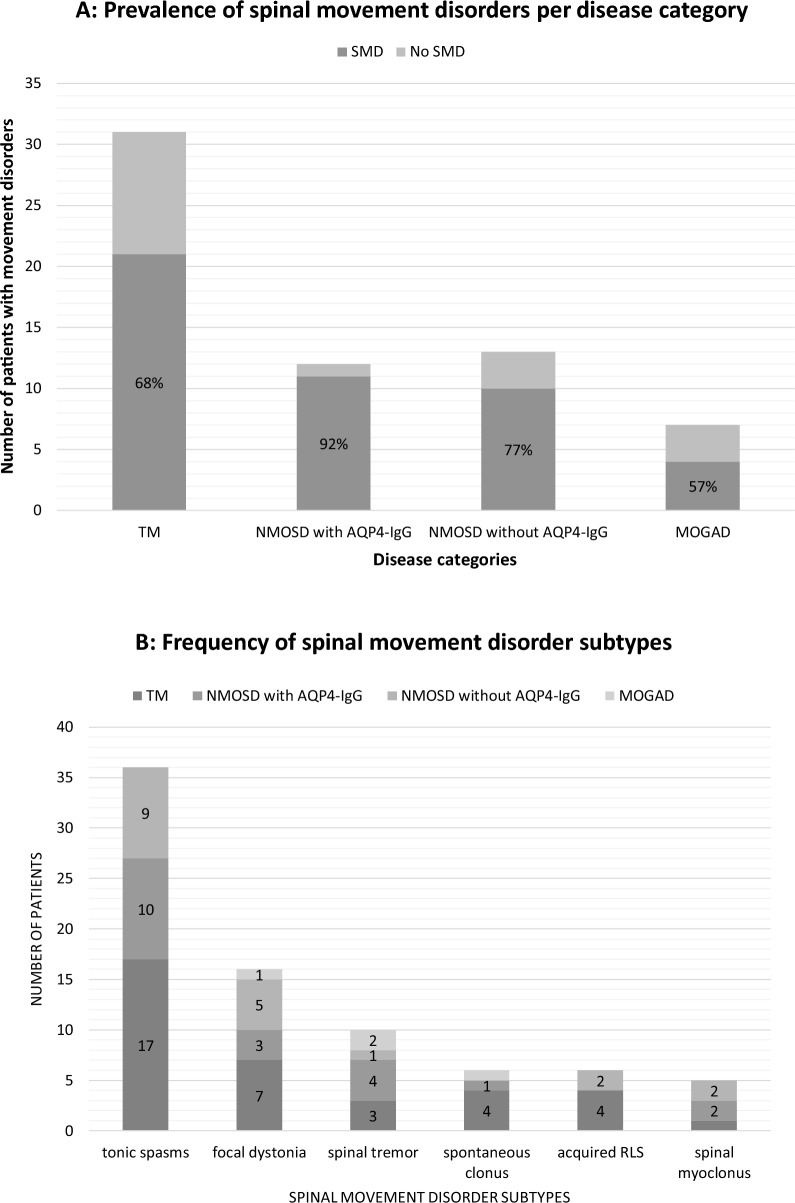
Comparison of spinal movement disorders by diagnosis: **A** number of patients with spinal movement disorders per disease category. **B** Number of patients with each spinal movement disorder subtype

From the spinal movement disorder standpoint (Fig. 2), NMOSD patients were more likely to have tonic spasms (*P* = 0.014, RR 1.699, 95% CI [1.120–2.576]), especially isometric tonic spasms (*P* = 0.044, RR 1.754, 95% CI [1.017–3.024]) and spinal myoclonus (*P* = 0.022, RR 6.167, 95% CI [0.732–51.906]). They were also more likely to have pain associated with their spinal movement disorders (*P* = 0.005, RR 3.571, 95% CI [1.352–9.436]) and were more likely to receive symptomatic treatment with carbamazepine or oxcarbazepine (*P* = 0.003, RR 17.000, 95% CI [2.083–138.710]).

Most of the statistically significant differences between NMOSD and other patients persisted even when the analysis was restricted to NMOSD with AQP4-IgG patients except that they were no longer significantly associated with spinal myoclonus. As for the findings on axial spinal MRI, seropositive patients were more likely to have lateral lesions compared to other patients while seronegative NMOSD patients were more likely to have central lesions.

MOGAD patients were less likely than other patients to have tonic spasms (*P* = 0.0016, RR 0.0976, 95% CI [0.0066 to 1.4391]) especially isometric tonic spasms (*P* = 0.0142, RR 0.1250, 95% CI [0.0084 to 1.8532]).

Patients with ITM and those with NMOSD without AQP4-IgG did not have any statistically significant association with or protection against any particular spinal movement disorder phenotype. Focal dystonia, spinal tremor, clonus, and RLS were statistically comparable in all diagnosis categories.

### Comparison between patients with and without spinal movement disorders (Table 3)

**Table 3:** comparison between patients with and without spinal movement disorders

	Patients with SMDs (*n* = 46)	Patients without SMDs (*n* = 17)	*P* value	Relative risk/mean difference	95% CI
Diagnosis					
AQP4 + ve	11 (23.9)	1 (5.9)	0.155 (by Fisher’s exact test)	1.336	1.038 to 1.719
MOG + ve	4 (8.7)	3 (17.6)	0.375	0.762	0.394 to 1.473
Seronegative	10 (21.7)	3 (17.6)	1.000	1.068	0.757 to 1.507
ITM	21 (45.7)	10 (58.8)	0.353	0.867	0.640 to 1.176
Age	50.1 ± 13.4	43.6 ± 14.2	0.099	6.477	− 1.258 to 14.212
Sex			0.757	1.094	0.803 to 1.490
M	14 (30.4)	4 (23.5)			
F	32 (69.6)	13 (76.5)			
Ethnicity			0.095	1.357	0.896 to 2.055
W	33 (71.7)	9 (52.9)			
AA	11 (23.9)	8 (47.1)			
Other	2 (4.3)	0 (0)			
Disease Duration	71.02 ± 108.7	10.53 ± 12.8	** < 0.001**	60.492	27.660 to 93.325
Follow up Duration	15.4 ± 14.9	8.4 ± 8.9	**0.029 (by Fisher’s exact test)**	6.980	0.763 to 13.196
Number of Visits	4.2 ± 3.2	2.7 ± 1.8	0.077	1.490	− 0.165 to 3.145
Cervical lesion	32 (69.6)	13 (76.5)	0.757	0.914	0.671 to 1.246
Thoracic lesion	30 (65.2)	10 (58.9)	1.000	1.000	0.734 to 1.363
Conus lesion	4 (8.7)	0 (0)	0.564	1.422	0.171 to 11.79
LETM	31 (67.4)	5 (29.4)	**0.009**	1.538	1.061 to 2.229
Lesion length	5.05 ± 4.68	3.67 ± 4.12	0.318	1.382	− 1.365 to 4.129
Anterior Cord	18 (39.1)	3 (17.6)	0.088	1.321	0.984 to 1.774
Posterior Cord	18 (39.1)	4 (23.5)	0.210	1.227	0.906 to 1.663
Lateral cord	13 (28.3)	1 (5.9)	0.084	1.409	1.089 to 1.822
Central cord	15 (32.6)	4 (23.5)	0.437	1.140	0.834 to 1.559
Corticosteroids	36 (78.3)	12 (70.6)	0.523	1.125	0.759 to 1.667
Plasmapharesis	21 (45.7)	4 (23.5)	0.111	1.277	0.959 to 1.700
Long-term IS or DMT	29 (63.0)	6 (35.3)	**0.049 (by Fisher’s exact test)**	1.365	0.977 to 1.906

Bold text indicates statistically significant differences between patients with and without spinal movement disorders

*SMDs* spinal movement disorders, *W* white, *AA* African American, *LETM* longitudinally extensive transverse myelitis, *IS* immunosuppressant, *DMT* disease-modifying therapy

Patients who developed spinal movement disorders, compared to those who did not, were more likely to have LETM (*P* = 0.009, RR 1.538, 95% CI [1.061–2.229]), longer disease duration (*P* = 0.000, mean difference = 60.492, 95% CI [27.660–93.325]), longer follow-up duration (*P* = 0.029, mean difference = 6.980, 95% CI [0.763–13.196]), and more likely to be treated with long-term disease-modifying therapy (*P* = 0.049, RR 1.365, 95% CI [0.977–1.906]). Patients with spinal movement disorders were mostly white (71%).

In the multivariate logistic regression model, LETM and AQP4-IgG seropositivity were independent predictors for the development of spinal movement disorders (*P* = 0.022, RR 6.416, 95% CI [1.308–31.474]; *P* = 0.022, RR 43.670, 95% CI [1.723–1107.0], respectively), while MOG-IgG and African American race were associated with a lower risk of developing these movement disorders (*P* = 0.022, RR 0.023, 95% CI [0.001–0.580]; *P* = 0.045, RR 0.151, 95% CI [0.024–0.961], respectively). Lesion location on axial MRI (i.e., central vs anterior vs lateral) was not predictive of the development of spinal movement disorders.

## Discussion

We conducted a prospective observational study focusing on spinal movement disorders in non-MS demyelinating disorders targeting the spinal cord. In this cohort with predominant LETM, spinal movement disorders were common and were present in all disorders. The prevalence rate in this cohort (73%) was much higher than the prevalence of spinal movement disorders in our previous prospective cohort that mainly consisted of patients with early MS (50%) [1]. This is likely due to the comparatively less extensive inflammatory damage of the motor tracts in MS myelitis [15]. Acquired RLS, the most common spinal movement disorder in MS [1], was rare in our cohort, likely because sensory-predominant myelitis is not as common in non-MS demyelinating disorders of the spinal cord compared to MS. Among the four diagnoses included in this study, NMOSD with AQP4-IgG, NMOSD without AQP4-IgG, MOGAD, and ITM, patients with AQP4-IgG had the highest prevalence (92%) of spinal movement disorders and were more likely to have tonic spasms and experience pain with their involuntary movements. The prevalence rate in NMOSD with AQP4-IgG in this study was much higher than what we found in a previous retrospective study (43%) [7] emphasizing how retrospective studies can underestimate involuntary movements and other NMOSD symptoms due to documentation bias in favor of details relating to relapses and immunotherapy. In addition, some spinal movement disorders may be overlooked or mislabeled in medical charts (e.g., labeling isometric spasms as “cramps”, paroxysmal dystonia as “spasms”, and RLS as “paresthesia”). Most previous studies of spinal movement disorders in NMOSD focused on tonic spasms alone and prevalence rates in these studies were also much lower than our tonic spasm prevalence rates of 83% in seropositive and 69% in seronegative patients. In a relatively large retrospective study from China, Li et al. reported a prevalence rate of 43% [4], which was higher than the 24% prevalence rate reported by Liu et al. in another Chinese study [3]. In one of the earliest studies on this topic from South Korea, Kim et al. reported a 25% prevalence rate of tonic spasms in NMOSD with AQP4-IgG, which was much higher than control samples of MS and ITM patients [2]. The difference in prevalence rates between our study and those studies are likely related to their retrospective nature and the lack of specific evaluation by a movement disorder specialist. However, racial differences may also be contributing to the difference in prevalence rates between studies. In our study, African American race was an independent protective factor against development of spinal movement disorders. The same finding was present in our previous retrospective study of spinal movement disorders in NMOSD [7] suggesting that racial differences may play a role in spinal movement disorder susceptibility. Therefore, this may also be contributing to the difference in prevalence rates between our predominantly white cohort and the Asian studies.

In addition to the different prevalence rates, our study had several other differences compared to previous studies. Spinal movement disorders in our cohort were mostly painless, and pain was almost exclusive to patients with tonic spams. Even within patients with tonic spasms, more than half the patients reported that their spasms were painless. In the studies by Li, Liu, and Kim, all patients reported pain associated with their spasms. Again, it is not clear if this is a biological difference between races or simply a documentation bias because of the predominantly retrospective nature of those studies. It is notable that in the study by Li et al., pain was mostly encountered in patients with complex tonic spasms, which was the most prevalent tonic spasm phenotype in their cohort but was very rare in ours. When considering all spinal movement disorder phenotypes, our study showed a longer interval (4.7 months) between relapse and movement onset compared to what is typically reported for tonic spasms in other studies (about 1 month). That is likely because tonic spams can occur as a relapse symptom or shortly after while other spinal movement disorder phenotypes like tremor or RLS can take a longer time to develop.

There are also many similarities between our study and previous work. Similar to our findings, Li et al. found that flexor spasms occurred mostly in the upper extremity while extensor spams mainly involved the lower extremity, concordant with the pyramidal distribution that favors increased tone in antigravity muscles after neurologic injury. They also found a higher prevalence of isometric spasms in truncal muscles similar to our findings. Our treatment response rate of 57% was comparable to their rate of 66% in patients receiving symptomatic treatment for spinal movement disorders highlighting the need for more effective symptomatic options for this impactful group of symptoms. In our cohort, some patients responded to traditional symptomatic therapies for spinal movement disorders and some did not. There was no clear signal favoring one treatment modality over others. Most patients with spinal movement disorders in previous studies reported a significant negative impact on their quality of life and our study showed that this impact is not restricted to pain and can extend to interference with walking and other activities of daily living.

We evaluated predictors of spinal movement disorders in patients with spinal cord demyelination and found that the presence of LETM was an independent predictor, likely due to the extensive inflammation and involvement of the motor and spinocerebellar tracts in longitudinal lesions. Unexpectedly, the actual length of the myelitis lesion (as represented by the number of vertebral levels) and the location of inflammation on axial MRI cuts were not predictive of the development of spinal movement disorders. Li et al. also did not find lesion length and location predictive of tonic spasms in their cohort. Although ephaptic transmission of neuronal impulses among demyelinated fibers is the proposed mechanism behind most paroxysmal spinal movement disorders [16], it remains unclear as to which tracts are involved in the production of the different hyperkinetic spinal movement disorder phenotypes. The pyramidal tracts are presumed to be the origin of tonic spasms, focal dystonia, and clonus [17–20], whereas the spinocerebellar tracts are presumed to be the origin of spinal tremor. However, the lack of correlation between the development of spinal movement disorders and the location of inflammation on axial MRI argues against the accuracy of these presumed localizations. Although we did not correlate lesion location to each spinal movement disorder phenotype, we expected that anterolateral lesions containing the motor tracts will be predictive of spinal movement disorders since tonic spasms and focal dystonia were most prevalent. Larger studies with more advanced MRI techniques may be needed to elucidate the exact anatomical generators of spinal movement disorders within the spinal cord.

Serological markers seem to play an important role in the susceptibility to spinal movement disorders with AQP4-IgG being a risk factor and MOG-IgG being associated with a lower risk regardless of the presence or absence of LETM. The link between tonic spasms and seropositivity to AQP4-IgG was shown in other studies [21] and it might be related to the pathophysiology of astrocyte-targeting AQP4 antibodies and their association with necrotizing inflammation in water channel-rich areas in the spinal cord. A European survey-based study and a Chinese retrospective study both demonstrated lower frequency of tonic spasms in MOGAD compared to NMOSD [22, 23]. Our prospective movement-focused study confirms this preliminary observation. The reason why MOG-IgG may be associated with a lower risk of spinal movement disorders in myelitis patients is unclear and likely reflects the pathophysiological differences between the two conditions. The primary demyelinating pathology and the improved remyelination in MOGAD compared to NMOSD may explain this finding.

Our study has several limitations. The rarity of NMOSD and MOGAD, and the recruitment of patients seen by a single provider resulted in a small number of patients in each diagnosis category. This limits the generalizability of the results and calls for caution when interpreting our findings. Given the tertiary referral nature of our center, a degree of selection bias towards more severe cases with higher frequency of spinal movement disorders may have occurred. Like most movement disorders, the differentiation between the various spinal movement disorder phenotypes was subjective and not based on electrophysiological or biomarker-based data. We tried to reduce the subjectivity of phenomenology rating by obtaining anonymous video samples that were evaluated by blinded raters. However, not all spinal movement disorders were present on exam during clinical visits and many spinal movement disorders were not video-recorded. Therefore, there might have been a degree of phenomenology misclassification, as it is the case in many studies of movement disorders. Our study was not designed to detect therapeutic responses and we could not determine a significant difference in treatment response between muscle relaxants, carbamazepine/oxcarbazepine, and botulinum toxin injections. Although most of the phenomenologies described in this paper have well-established spinal basis based on previous studies in demyelinating diseases from our group and others, we cannot confirm the spinal origin of the movement disorders in patients with coexisting brainstem lesions with absolute confidence. Overall, the number of patients with isolated brainstem lesions unrelated to extension from high cervical myelitis was low. Lastly, although we tried to identify anatomical generators of spinal movement disorders based on axial MRI cuts, we only utilized standard MRI techniques and conventional sequences. Future studies utilizing advanced MRI techniques including higher field or myelin-based imaging may be better suited to identify anatomical generators of spinal movement disorders.

## Conclusion

Spinal-generated movement disorders are highly prevalent in non-MS demyelinating disorders of the spinal cord and their prevalence rates are much higher than the rates reported in MS. Although this was true about all four diagnostic categories evaluated, the prevalence was highest in NMOSD with AQP4-IgG and lowest (approaching the same MS prevalence) in MOGAD. Our prospective movement-focused study revealed a much higher prevalence rate of spinal movement disorders in NMOSD than previous studies and a lower rate of associated pain. Tonic spasms, focal dystonia, spinal tremor, and spinal myoclonus were seen more frequently in this cohort than MS but RLS and other sensory-related movement disorders were less frequent. Spinal movement disorders can happen independent of and in absence of spasticity. In addition to pain, spinal movement disorders have several other negative impacts on quality of life, including interference with ambulation and balance. Amongst these four diseases, LETM and AQP4-IgG are independent risk factors for spinal movement disorders, while MOG-IgG and African American race are associated with a lower risk. Close to half the patients do not respond to symptomatic therapy including carbamazepine/oxcarbazepine, and there is a pressing need for better symptomatic treatments for spinal movement disorders. Future studies should utilize advanced MRI techniques to detect the precise anatomical generators of the different spinal movement disorder phenotypes and employ randomized controlled trials to identify evidence-based therapies for spinal movement disorders.

## Supplementary Information

Below is the link to the electronic supplementary material.

Video 1: Right ankle dorsiflexor spasms in a patient with NMOSD with AQP4-IgG and cervicothoracic myelitis. (MOV 42457 KB)

Video 2: Right finger rest and postural spinal tremor with distal dystonic posturing of the fingers in a patient with NMOSD without AQP4-IgG and cervical myelitis. (MOV 25905 KB)

Video 3: Bilateral upper extremity irregular rest/postural/kinetic tremor in a MOGAD patient with upper cervical myelitis extending to the brainstem. (MP4 11713 KB)

Video 4: Adductor hip tonic spasms in a patient with ITM. (MP4 5293 KB)

Supplementary file5 (DOCX 13 KB)

## Data Availability

Source data can be made available for any qualified investigator.
