# Supplementary material for: Spinal movement disorders in NMOSD, MOGAD, and idiopathic transverse myelitis: a prospective observational study
Source: J Neurol. 2024 Jul 8;271(9):5875–85. doi: 10.1007/s00415-024-12527-6 (PMC11377660; doi:10.1007/s00415-024-12527-6)
Supplement: Supplementary file 5 — Supplementary file5 (DOCX 13 KB) [file 415_2024_12527_MOESM5_ESM.docx]

**Movement disorder survey for patients with demyelinating diseases:**

*Has the patient experienced any of the following movement disorders at any point in their lifetime?*

Spasticity:

Tonic spasms:

- Isometric:
- Extensor:
- Flexor:
- Adductor/inversion:
- Complex:

Tremor:

- Action:
- Resting:
- Intention:
- Other:

RLS:

Focal dystonia:

- Paroxysmal:
- Fixed:

Other movement disorders:

- Myoclonus:
- Spontaneous clonus:
- Pseudoathetosis:
- Hyperekplexia:
- Fasciculation:
- Parkinsonism:
- Chorea:
- Balism:

For each movement disorder comment on date of onset, relation of onset to onset of the demyelinating disease, interval from relapse, periodicity, frequency, triggers, association with pain, other impact, body part involved, and response to treatment.
